# Cardiovascular risk in patients with a relationship with oxidative stress and dyslipidemia

**DOI:** 10.5937/jomb0-52038

**Published:** 2025-06-13

**Authors:** Vesna Karanikolic, Mirjana Bakic, Sanja Gluscevic, Filiz Mercantepe, Aleksandra Klisic

**Affiliations:** 1 University of Nis-School of Medicine, Clinic for Skin Diseases of the Clinical Center Nis, Nis; 2 Clinical Center of Montenegro, Clinic for Dermatovenerology, Podgorica, Montenegro; 3 Clinical Center of Montenegro, Department of Neurology, Podgorica, Montenegro; 4 Recep T ayyip Erdogan University, Faculty of Medicine, Department of Endocrinology and Metabolism, Rize, Turkey; 5 University of Montenegro-Faculty of Medicine, Podgorica, Montenegro; 6 Primary Health Care Center, Center for Laboratory Diagnostics, Podgorica, Montenegro

**Keywords:** cardiovascular risk, inflammation, oxidative stress, psoriasis, kardiovaskularni rizik, zapaljenje, oksidativni stres, psorijaza

## Abstract

**Background:**

Patients with psoriasis are at an increased risk of cardiovascular disease (CVD). Psoriasis and atherosclerosis share the common soil of inflammation and oxidative stress in their pathogenesis. The current study aimed to examine cardiovascular risk concerning some non-traditional (i.e., biomarkers of oxidative stress and inflammation) and traditional metabolic parameters in patients with psoriasis.

**Methods:**

A total of 68 (57% men) patients with psoriasis were included. Traditional metabolic parameters, markers of oxidative stress[i.e., oxidation protein products (AOPP), malondialdehyde (MDA), catalase (CAT), and superoxide dismutase (SOD)] and inflammation (C-reactive protein) were measured. The atherosclerotic cardiovascular disease (ASCVD) risk score was used to measure CVD risk. Patients were divided into ASCVD score tertiles.

**Results:**

Patients with a higher ASCVD score had significantly lower high-density lipoprotein cholesterol (HDL-C), higher triglycerides (TG), and higher TG/HDL-C ratio (p for trend p<0.001). Among redox status parameters, only AOPP showed a significant increase in parallel with the ASCVD score increase (p=0.011). In univariate binary logistic regression analysis, AOPP [OR, 95% CI=1.027 (1.004-1.051), p=0.021] and TG [OR, 95% CI =7.220 (2.041-25.548), p=0.002] correlated with the ASCVD risk score. In multivariate analysis (backward method), only TG was an independent predictor of ASCVD score [OR, 95%CI =7.220 (2.041-25.548), p=0.002].

**Conclusions:**

The results show the association between ASCVD score and oxidative stress (AOPP) and dyslipidemia (TG), respectively, in patients with psoriasis, but only TG retained its independent association with ASCVD risk score. Measuring serum TG levels is very important in patients with increased ASCVD risk concerning psoriasis.

## Abstract (sr)

**Uvod:**

Pacijenti sa psorijazom imaju povećan rizik od kardiovaskularnih bolesti (KVB). Psorijaza i ateroskleroza dele zajedničko tlo zapaljenja i oksidativnog stresa u svojoj patogenezi. Ova studija ima za cilj da ispita kardiovaskularni rizik u odnosu na neke netradicionalne (tj. biomarkere oksidativnog stresa i upale) i tradicionalne metaboličke parametre kod pacijenata sa psorijazom.

**Metode:**

U studiju je uključeno ukupno 68 pacijenata (57% muškaraca) sa psorijazom. Mereni su tradicionalni metabolički parametri, markeri oksidativnog stresa (tj. produkti uznapredovale oksidacije proteina (AOPP), malondialdehid (MDA), katalaza (CAT), superoksid dizmutaza (SOD)) i zapaljenja (C-reaktivni protein). Za merenje rizika od KVB je korišćen skor rizika od aterosklerotičnih kardiovaskularnih bolesti (ASCVD). Pacijenti su, potom, podeljeni prema tercilnim vrednostima ASCVD skora.

**Rezultati:**

Pacijenti sa višim ASCVD skorom su imali značajno nižu koncentraciju holesterola u lipoproteinima velike gustine (HDL-C), više vrednosti triglicerida (TG) i veći odnos TG/HDL-C (p<0,001). Među parametrima redoks statusa, samo su produkti uznapredovale oksidacije proteina (AOPP) pokazali značajno povećanje paralelno sa povećanjem ASCVD skora (p=0,011). U univarijantnoj binarnoj logističkoj regresionoj analizi AOPP [OR, 95%CI= 1,027 (1,004-1,051), p=0,021] i TG [OR, 95%CI= 7,220 (2,041-25,548), p=0,002] su bili u korelaciji sa ASCVD skorom. U multivarijantnoj analizi je samo TG bio nezavisni prediktor ASCVD skora [OR, 95% CI=7,220 (2,041-25,548), p=0,002], dok je AOPP izgubio svoj prediktivni kapacitet.

**Zaključak:**

Rezultati ovog istraživanja pokazuju povezanost ASCVD skora i oksidativnog stresa (AOPP) i dislipidemije (TG) kod pacijenata sa psorijazom, ali su samo vrednosti TG nezavisno povezane sa ASCVD skorom. Određivanje nivoa TG u serumu je od velikog značaja kod pacijenata obolelih od psorijaze, a sa povećanim rizikom od ASCVD.

## Introduction

Psoriasis is a multisystemic auto-inflammatory disorder characterized by a complex network of environ mental factors and genetic background [1] [2]. The prevalence of psoriasis varies from nearly 12% [1] to nearly 12% in Caucasians [3].

It is assumed that an unknown autoantigen or cells of innate immunity are the instigators of psoriasis that trigger T cells and T-helper 1 (Th1) cytokines [4]. Stimulating atypical Th1 and Th17 lymphocytes with concomitant proinflammatory cytokine secretion is a main feature of psoriasis [5]. The exaggerated inflammatory cascade further promotes the production of reactive oxygen species (ROS), aggravates oxidative stress, and concomitantly diminishes antioxidative defence, leading to cell destruction [6].

The vicious circle between oxidative stress and inflammation represents the common soil not only for psoriasis pathogenesis but also for related comorbidities, such as obesity, liver steatosis, diabetes type 2, and hypertension, which are all independent risk factors for cardiovascular disease (CVD) [3] [7].

To our knowledge, no studies investigated CVD risk in patients with psoriasis in Montenegro. Since CVD represents the leading cause of mortality in this country [8], and considering the mutual prooxidant and proinflammatory nature of psoriasis and athero sclerosis [4], it is of utmost importance to enlighten the mechanisms of these two entities. Hence, the current study aimed to examine CVD risk concerning some non-traditional (i.e., biomarkers of oxidative stress and inflammation) and traditional metabolic parameters in patients with psoriasis since this issue is not well-explored.

The similar Th1 and Th17 cell-mediated immune mechanisms are the underlying pattern of psoriasis and atherosclerosis [4]. Interestingly, psoriatic and atherosclerotic plaques comprise similar proinflammatory cytokines, cell infiltrates, growth factors, and adhesion molecules [4]. Indeed, it has been shown that patients with psoriasis are at an increased risk of CVD [2]. However, such issue is often neglected in primary care settings, and individuals with psoriasis are often underrecognised and undertreated concerning related concomitant CVD risk factors [3]. Hence, it is important to estimate the CVD risk in patients with psoriasis on time and identify high-risk groups that benefit from adequate therapeutic strategies [1] [9]. In line with this, different CVD risk scores were validated as estimators of the probability of a person experiencing a major cardiovascular event in the next ten years [9]. Additionally, some novel proinflammatory biomarkers that could provide deeper insight into the pathophysiological mecha nisms of psoriasis are emerging [5] [10] and could represent a reliable tool for estimating CVD risk in those individuals.

## Materials and methods

Phlebotomy was performed in the morning after 8 hours of fasting, as described previously [6] [10]. Cardiometabolic parameters, i.e. C-reactive protein (CRP), triglycerides (TG), total cholesterol (TC), low-density lipoprotein cholesterol (LDL-C), high-density lipoprotein cholesterol (HDL-C), glucose, uric acid, creatinine, urea, aspartate aminotransferase (AST), and alanine aminotransferase (ALT) were determined on chemistry analyser Roche Cobas c501 (Roche Diagnostics GmbH, Mannheim, Germany) using routine commercial methods, i.e., standardised [8].

Oxidative stress biomarkers were determined using methods validated in our laboratory [6]. Malondialdehyde (MDA) was measured following the analysis of thiobarbituric acid reactive substances by the thiobarbituric acid test [11]. Advanced oxidation protein products (AOPP) were determined according to Witko-Sarsat’s method recommendations by a reaction with glacial acetic acid and potassium iodide [12]. Superoxide dismutase (SOD) activity was determined by the method of Misra and Fridovich [13]. Catalase (CAT) activity was determined following the liberation of oxygen from hydrogen peroxide (H_2_O_2_), following the formation of the complex with ammonium molybdate [14].

### Patients and methods

### Patients

After obtaining the approval of the Institutional Review Board, we conducted a study following the Helsinki Medical Declaration related ethical principles. A total of 68 patients (39 men (57%) and 29 women (43%)) with psoriasis with a median disease duration of 8.4 (5.7–16.1) years who voluntarily accepted to participate in the study were included. These outpatient participants were included during their regular check-ups in the Clinic for Dermato venerology, Clinical Center of Montenegro, once psoriasis diagnosis was established.

Each patient signed an informed consent form and filled in the questionnaire. The patients underwent anthropometric measurements and blood pressure the same morning when blood sampling was performed.

Thirty-eight (55.9%) patients used local therapy, and 30 (44.1%) used methotrexate. Patients who used biologic therapy, psoriasis patients with CVD, stroke, malignancies, mental disturbances, autoimmune diseases, skin diseases other than psoriasis, pregnant women, and those who used antioxidant supplements were excluded from the study.

The Psoriasis Area and Severity Index (PASI) was used to display the severity of psoriasis [6].

### Calculation of scores

The Oxy score was calculated by subtraction of the protective score (i.e., Antioxidant score, which was obtained as an average of standardized antioxidant variables (SOD and CAT)) from the damage score (i.e., Prooxidant score, which was obtained as the average of standardized prooxidant factors (i.e., AOPP and MDA)) [15] [16].

The atherosclerotic cardiovascular disease (ASCVD) risk score was used as a measure of CVD risk since the American Heart Association (AHA) and the American College of Cardiology (ACC) guidelines showed good accuracy of this algorithm in predicting cardiovascular events [9].

The ASCVD risk score was determined by incorporation of the following variables into its calculation: race, gender, age, smoking status, HDL-C, LDL-C, TC, systolic (SBP) and diastolic blood pressure (DBP), treatment of hypertension, aspirin treatment, use of statins, and history of diabetes [9].

### Statistical analysis

The SPSS statistical package (version 18.0 for Windows, SPSS, Chicago, IL, USA) was used for the statistical analysis. The data was presented as counts and percentages for categorical variables or as median (interquartile range) for continuous variables. The data distribution was checked by using the ShapiroWilks test. A Kruskal-Wallis test was used for the three groups’ comparison (with the Mann-Whitney U test as a post-hoc test). Spearman’s nonparametric correlation was used to determine the correlation between ASCVD and examined parameters. Binary logistic regression analysis with univariate and multivariate modality was used for significant predictors of ASCVD score high values selection (third vs. first tertile value). A p level <0.05 was considered statistically significant.

## Results

The general data about patients and redox status parameters are presented in Table 1. Patients with higher ASCVD scores were significantly older, with a higher percentage of men and smokers. Also, patients with higher ASCVD scores were substantially more likely to have lower HDL-C, higher TG, and higher TG/HDL-C ratios, representing the predominance of small, dense LDL particles in their circulation. Along with all already mentioned lipid status disturbances, it was obvious that the same regularity is true for non-HDL-C. Among redox status parameters, only AOPP showed a significant increase parallel to the ASCVD score increase.

**Table 1:** Basic clinical, anthropometric and biochemical data according to ASCVD score’s tertile value subgroups. Legend: ASCVD score, atherosclerotic cardiovascular disease risk score; BMI, body mass index; TC, total cholesterol; LDL-c, low-density lipoprotein cholesterol; HDL-c, high-density lipoprotein cholesterol; TG, triglycerides; AST-aspartate aminotransferase; ALT-alanine aminotransferase; CRP, C-reactive protein; AOPP-advanced oxidation protein products; MDA, malondialdehyde; SOD, superoxide dismutase; CAT, catalase; PASI score, psoriasis area and severity index.<br>P from Kruskal-Wallis test; *, **, *** P<0.05, 0.01, 0.001 vs. first tertile group; ^#^, ^##^P<0.05, 0.01 vs. second tertile group

Parameter	First tertile<br><3.2	Second tertile<br>3.3–9.9	Third tertile<br>>10	P
ASCVD score				
Age, years	43.0 (30.0–51.5)	55.0 (46.0–60.0)***	66.0 (58.5–68.5)***,^###^	< 0.001
Gender (m/f), (n/%)	6/16 (15/55)	17/6 (44/21)	16/7 (41/24)	0.001
Smoking (no/yes)	21/2 (42/10)	16/8 (32/40)	13/10 (26/50)	0.027
BMI, kg/m^2^	26.0 (23.9–28.1)	28.0 (24.9–29.6)	27.7 (25.3–31.1)	0.210
Glucose, mmol/L	5.2 (4.9–5.6)	5.6 (5.1–6.3)*	5.4 (5.0–6.2)	0.147
Urea, mmol/L	4.6 (3.7–5.2)	5.2 (4.1–6.2)	5.2 (4.5–6.7)	0.280
Creatinine, μmol/L	63 (60–77)	70 (56–80)	70 (60–84)	0.222
Uric acid, μmol/L	254 (231–340)	308 (231–335)	310 (266–351)	0.160
TC, mmol/L	4.92 (4.51–6.06)	5.32 (4.56–5.98)	5.55 (4.73–6.57)	0.317
LDL-C, mmol/L	2.83 (2.30–3.63)	3.18 (2.62–3.62)	3.46 (2.21–4.08)	0.617
HDL-C, mmol/L	1.5 (1.4–1.9)	1.3 (1.0–1.7)*	1.2 (0.9–1.5)***	<0.001
TG, mmol/L	0.99 (0.81–1.38)	1.48 (1.08–2.00)*	2.12 (1.50–2.76)***,^#^	<0.001
TG/HDL-C	0.64 (0.51–0.90)	1.06 (0.72–1.68)**	1.69 (1.11–3.04)***,^#^	<0.001
Non-HDL-C, mmol/L	3.31 (2.71–4.34)	3.94 (3.41–4.32)	4.38 (3.48–5.15)*	0.074
AST, U/L	22 (20–26)	20 (18–26)	25 (19–35)	0.250
ALT, U/L	22 (18–36)	26 (19–35)	24 (18–36)	0.833
CRP, mg/L	0.95 (0.52–2.96)	1.53 (0.79–2.59)	2.10 (0.65–4.55)	0.206
AOPP, μmol/L	65 (67–75)	67 (60–81)	97 (68–105)**,^#^	0.011
MDA, μmol/L	4.62 (4.03–5.16)	4.59 (3.44–5.47)	4.31 (2.02–8.23)	0.935
SOD, U/L	9.2 (9.0–9.4)	9.3 (9.3–9.4)	9.3 (9.2–9.4)	0.308
CAT, U/L	0.486 (0.246–0.637)	0.412 (0.234–0.652)	0.497 (0.308–0.686)	0.670
Prooxidant score	-0.10 (-0.75–0.81)	0.23 (-0.89–0.92)	0.11 (-1.19–2.85)	0.864
Antioxidant score	0.61 (-0.47–1.30)	0.28 (-0.53–1.37)	0.67 (-0.19–1.53)	0.670
Oxy score	-0.10 (-1.29–0.90)	-0.03 (-1.49–0.92)	-0.66 (-1.90–1.66)	0.975
PASI score	12 (10–17)	16 (15–18)	15 (10–18)	0.316

**Figure 1:**
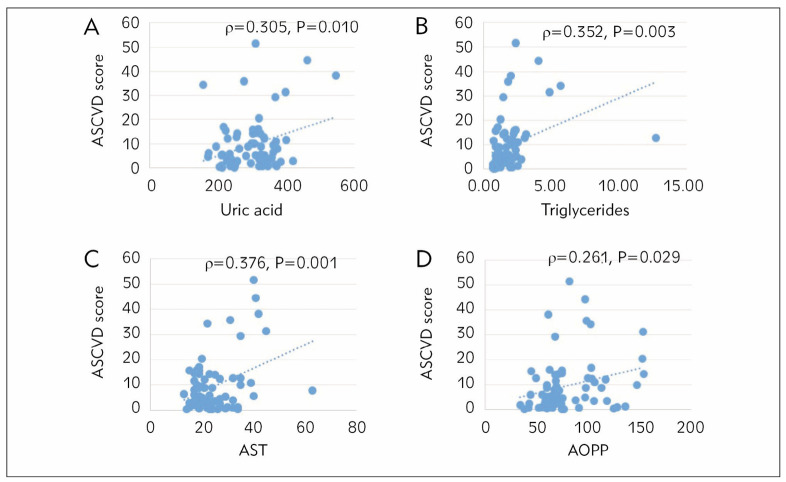
Spearman’s nonparametric correlation between ASCVD score and uric acid (Figure 1A), TG (Figure 1B), AST (Figure 1C), and AOPP (Figure 1D) concentrations.

Figure 1 presents significant correlations between the ASCVD score and other measured parameters, i.e., uric acid, TG, AST, and AOPP. Other measured and tested parameters did not significantly correlate with the ASCVD score (data not shown here).

The following statistical analysis was binary logistic regression analysis for high ASCVD values prediction (third vs first ASCVD tertile value), firstly univariate and after that multivariate analysis of parameters which had P 0.100 in univariate analysis. The results are presented in Table 2.

**Table 2:** Univariate and multivariate binary logistic regression analysis for high ASCVD score values prediction (above 75^th^ percentile). Legend: BMI, body mass index; TG, triglycerides; AST, aspartate aminotransferase; ALT, alanine aminotransferase; CRP, C-reactive protein; AOPP, advanced oxidation protein products; MDA, malondialdehyde; SOD, superoxide dismutase; CAT, catalase; PASI score, psoriasis area and severity index; SE, standard error, OR, odds ratio (95th CI, confidence interval); P from the binary logistic regression analysis.

Factors (univariate analysis)	B (SE)	Wald coefficient	OR (95% CI)	P
BMI, kg/m^2^	0.117 (0.078)	2.242	1.124 (0.965–1.309)	0.134
Urea, mmol/L	0.389 (0.310)	1.576	1.475 (0.804–2.708)	0.209
Creatinine, μmol/L	0.033 (0.022)	2.213	1.033 (0.990–1.079)	0.137
Uric acid, μmol/L	0.006 (0.004)	2.201	1.006 (0.998–1.015)	0.138
TG, mmol/L	1.977 (0.645)	9.401	7.220 (2.041–25.548)	0.002
AST, U/L	0.074 (0.041)	3.329	1.077 (0.995–1.166)	0.068
ALT, U/L	0.001 (0.018)	0.003	1.001 (0.967–1.037)	0.957
CRP, mg/L	0.130 (0.107)	1.497	1.139 (0.925–1.404)	0.221
AOPP, μmol/L	0.027 (0.012)	5.344	1.027 (1.004–1.051)	0.021
MDA, μmol/L	0.018 (0.051)	0.121	1.018 (0.922–1.124)	0.727
SOD, U/L	-0.178 (1.275)	0.019	0.837 (0.069–10.195)	0.889
CAT, U/L	0.946 (1.291)	0.537	2.576 (0.205–32.355)	0.464
PASI score	0.044 (0.082)	0.289	1.045 (0.891–1.226)	0.591
Factors (multivariate analysis)				
TG, mmol/L	1.977 (0.645)	9.401	7.220 (2.041–25.548)	0.002

The results of univariate analysis pointed out TG and AOPP as significant predictors of high ASCVD values and AST as a conditionally significant predictor (P=0.068). Those 3 parameters were included in multivariate analysis (backward method), and this part of the analysis highlighted TG as the only parameter left in the best model for ASCVD score prediction.

## Discussion

The current study’s findings show that increased oxidative stress (i.e., as determined by higher AOPP) and dyslipidemia (i.e., defined by higher TG levels) are significantly associated with a higher ASCVD score in patients with psoriasis. However, after further analysis, only TG levels retained the independent association with ASCVD.

This is the first study conducted in Montenegro that examined the ASCVD score in patients with psoriasis. CVD is the leading cause of mortality in Montenegro [8]. Given that the estimation of CVD risk is often neglected in primary care settings in this country (10), especially concerning psoriasis as an independent risk factor for CVD, such an issue requires a high level of attention. Recognising patients with increased ASCVD risk might enable physicians to implement treatment guidelines to prevent cardiovascular events in psoriasis [7].

The nexus between psoriasis and metabolic comorbidities such as obesity, metabolic syndrome, and type 2 diabetes has already been reported [3]. Since all mentioned metabolic disorders are independent risk factors for CVD, it is unsurprising that psoriasis and CVD share the common patho physiological mechanisms involving inflammation and oxidative stress as the typical features of these entities [3] [6] [10].

The impaired function of regulatory T cells and proinflammatory effects of macrophages play a significant role in atherogenesis and aggravate atheromatous plaque in patients with psoriasis [3]. The Th1 lymphocytes activate Th1 cytokines, T cells, and antigen-presenting cells in psoriasis. Elevated Th1 and Th17 lymphocytes were also found in atherosclerotic lesions in these patients. The increased release of proinflammatory cytokines by lymphocytes promotes endothelial dysfunction and atherosclerosis onset. Indeed, it was shown that plaques in psoriasis and atherosclerosis are composed of similar proinflammatory cytokines, angiogenic/growth factors, adhesion molecules, and chemokines [4].

The increased production of proinflammatory cytokines favours the synthesis of ROS, further contributing to atherosclerotic plaque generation through the promotion of macrophage activation by building up the oxidatively modified accumulated LDL. The macrophages are further transformed into foam cells, an important part of atherogenesis [17].

The progression of atherosclerotic plaques and the risk of its rupture resulting in thrombus formation is aggravated by prolonged comorbidity-related inflammation and oxidative stress [3]. In our previous study [5], we have shown for the first time higher indexes of platelet activation and reactivity, i.e., higher mean platelet volume-to-platelet ratio (MPR) and red cell distribution width-to-platelet ratio (RPR) in patients with psoriasis and comorbidities compared with patients without comorbidities, which could be explained by a prolonged effect of inflammation [18]. Moreover, a positive correlation between these indexes and oxidative stress biomarkers with atherosclerotic lesion complexity and severity was recently confirmed [18] [19].

Oxidative stress biomarkers have been suggested as a valuable and promising tool in addition to traditional biomarkers for better discrimination and stratification of patients with increased cardiometabolic risk and improved therapeutic strategies [20]. Still, almost none of these biomarkers has become a part of a routine panel of biomarkers in clinical practice [21].

We have recently shown the increased level of AOPP and CAT in patients with psoriasis vs. controls [6]. However, although we recorded a significant increase in AOPP levels in the highest ASCVD risk scores in patients with psoriasis in the current study, this oxidative stress biomarker did not retain its independent prediction in deeper statistical analysis. Such findings follow a previous prospective study that did not find an association between any of the investigated oxidative stress parameters and adverse cardiovascular events [22].

The independent association between ASCVD score and TG in the current study might be explained by the fact that prolonged higher TG levels in psoriasis-related cardiometabolic disturbances cause inflammation and vice versa [23]. An increased lipolysis of TG in adipose tissue following the aggravation of insulin resistance and increased secretion of free fatty acids promote higher levels of more atherogenic small dense LDL, increased synthesis of TG-rich very low-density lipoprotein (VLDL), decreased clearance of TG-rich lipoproteins and alterations in HDL composition and an enhanced clearance of HDL particles [24]. Therefore, it is assumed that decreased clearance of TG-rich lipoproteins and increased VLDL production in the liver contribute to the increased serum TG levels in the inflammatory response [23]. In line with this, increased serum VLDL and TG levels were recorded 2 h after the administration of the proinflammatory cytokines in the rat, and this increase persisted for at least 24 h after the treatment [25]. The alteration of biomolecules related to the metabolism of lipids further enhances inflammation and immune response in patients with psoriasis [1].

Based on the Third National Health and Nutritional Examination Survey data, hypertriglyceridemia was shown to have the strongest correlation with CVD risk compared with other metabolic syndrome components [26]. Additionally, TG levels were linked with incident cardiovascular events independently of other traditional risk factors and lipid parameters [27].

Given their increased CVD risk, the current study emphasizes the importance of screening patients with psoriasis for hypertriglyceridemia. Such cost-effective, routinely measured laboratory parameters are suggested to be an inevitable part of the diagnostic panel of biomarkers in patients with psoriasis.

The study’s limitation is the inability to apply imaging diagnostic procedures, such as carotidintima media thickness, as a surrogate marker of atherosclerosis, which could provide a deeper insight into CVD pathomechanisms. Also, our prospective validation of the ASCVD risk score in such patients was limited. Future studies with longitudinal design and larger sample sizes are needed to examine the causality of oxidative stress, inflammation, and dyslipidemia, respectively, and CVD in patients with psoriasis.

## Conclusion

The current study shows the association between the ASCVD score and oxidative stress (AOPP) and dyslipidemia (TG) in patients with psoriasis. However, only serum TG levels appeared to be independently associated with the ASCVD risk score. The screening of hypertriglyceridemia is of great importance in patients with increased CVD risk concerning this disease entity.

## Dodatak

### Conflict of interest statement

All the authors declare that they have no conflict of interest in this work.
